# Mediating Effect of Diabetes Mellitus on the Association Between Chromosome 9p21.3 Locus and Myocardial Infarction Risk: A Case-Control Study in Shanghai, China

**DOI:** 10.3389/fendo.2018.00362

**Published:** 2018-07-18

**Authors:** Zhijun Wu, Haihui Sheng, Xiuxiu Su, Xiang Gao, Lin Lu, Wei Jin

**Affiliations:** ^1^Department of Cardiology, Ruijin Hospital, Shanghai Jiao Tong University School of Medicine, Shanghai, China; ^2^National Engineering Center for Biochip at Shanghai, Shanghai, China; ^3^Department of Nutritional Sciences, Pennsylvania State University, State College, PA, United States

**Keywords:** mediation analysis, myocardial infarction, diabetes mellitus, genetic risk score, metabolic disease

## Abstract

**Background:** Previous genome-wide association studies revealed that the chromosome 9p21.3 locus is associated with an increased risk of myocardial infarction (MI) and diabetes mellitus (DM). However, it is unclear whether the 9p21.3-MI association is direct or mediated by pathways related to DM.

**Study Design:** We applied mediation analysis to examine the potential mediating effect of DM on the association between the 9p21.3 genetic risk score (GRS; ranged from 0 to 8) and MI in a case-control study of 865 MI patients and 927 controls without coronary artery disease (CAD). The GRS combining 4 lead 9p21.3 single nucleotide polymorphisms (rs1333040, rs4987574, rs2383207, and rs1333049) was constructed.

**Results:** Each 1 unit increase in weighted 9p21.3 GRS was associated with a 9% increased DM risk (95% confidence interval (CI) 1.00, 1.19) in the control group and a 14% increased MI risk (95% CI 1.09, 1.20). Mediation analyses yielded a direct-effect odds ratio (OR) of 1.06 (95% CI 1.04, 1.08) and an indirect-effect OR of 1.00 (95% CI 0.99, 1.01) for weighted GRS. DM mediated 4.40% (95% CI 3.42, 6.52) of the total association between the weighted GRS and MI risk. Individuals with the highest quartile of 9p21.3 GRS and DM had a 6-fold higher MI risk than those with the lowest quartile of 9p21.3 GRS and non-DM (OR 6.03, 95% CI 3.48, 10.5).

**Conclusion:** DM is a weak mediator that explains a small fraction of the 9p21. 3-MI association in Chinese adults. Nevertheless, there is a strong synergistic effect between DM and the 9p21.3 GRS on MI risk.

## Introduction

Myocardial infarction (MI) is a serious consequence of metabolic risk factors such as diabetes mellitus (DM) (1). The inheritance of MI and metabolic diseases is multifactorial, with genetic risk factors, environmental exposures and gene-environmental interactions all affecting the risk. Identifying the modifying, mediating, and confounding effects of metabolic risk factors may be beneficial for understanding the mechanisms underpinning atherosclerosis and in preventing the development of the disease.

Recent genome-wide association studies (GWASs) have consistently found that MI and DM may share genetic signatures, in addition to conventional risk factors (2). The chromosome 9p21.3 region is a common susceptibility locus for atherosclerotic cardiovascular diseases (CVD) manifestations (e.g., MI and coronary artery disease (CAD)), DM, and intracranial aneurysm (3–5). It also has clinical significance as a target for preventing atherosclerotic CVD and DM. This region does not contain any functional protein-coding genes, but expresses a long noncoding RNA *ANRIL* (antisense noncoding RNA in the inhibitor of CDK4 (INK4) locus) (6, 7). Although the exact function of *ANRIL* remains unclear, it is thought to be the main regulator of the neighboring genes, cyclin-dependent kinase 2A (*CDKN2A*) and 2B (*CDKN2B*). These 2 genes are tumor suppressors that have many effects on the cell cycle and vascular remodeling, such as regulating β-cell and vascular smooth muscle cell proliferation (8). Thus, it is possible that this locus may predispose individuals to both CAD/MI and DM through a common pathway. The magnitude of the 9p21.3-CAD/MI association varied according to the DM status or extent to glycemic control in diabetic people, suggesting that DM has a strong modifying effect on the gene-disease association (9, 10). However, these studies did not consider the intermediate DM-related pathway on the process underlying a 9p21.3-MI association. The extent of any additional contribution of DM beyond the direct pro-atherosclerosis effect of the 9p21.3 locus, which is operating through non-metabolic pathway, remains unclear (11).

In this study, we sought to quantify the mediating role of DM in the association between the 9p21.3 locus and MI in a Southern Chinese Han cohort. Second, we assessed the joint effect of the 9p21.3 locus and DM on the occurrence of MI. We genotyped 4 top associated single nucleotide polymorphisms (SNPs) within this region to represent the common characteristics of the 9p21.3 locus and constructed a genetic risk score (GRS) to increase statistical power (12–15).

## Methods

### Study participants

This is a case-control study that included 865 individuals with new-onset MI and 927 CAD-free controls who were living in Shanghai and undergoing cardiac catheterization at the Ruijin Hospital. All individuals were primarily of Southern Han Chinese ethnicity.

Information on socio-demographic characteristics (e.g., sex and age), cigarette smoking (non-smoker and smoker), drinking habits (non-drinker and drinker), and medical history (e.g., hypertension and diabetes) were collected by a questionnaire administered at the time of recruitment. Three experienced cardiologists reviewed all participants' medical records to confirm their clinical characteristics. MI was defined according to the ACC/AHA criteria based on positive cardiac enzymes, clinical symptoms, and typical electrocardiographic changes (16, 17). As previously described (18, 19), individuals with MI who had ≥50% stenosis or atherothrombosis in at least one major epicardial coronary artery or a main branch were included in our study.

Individuals with prior atherosclerotic CVD (e.g., CAD, MI or peripheral vascular disease), cardiomyopathy, congenital heart disease, pulmonary heart disease, valvular heart disease, infection, chronic kidney disease, tumor, or autoimmune disease were excluded from the case and control groups.

### SNP selection and genotyping

Overnight blood specimens were drawn from all the individuals. Genomic DNA was extracted from peripheral blood leukocytes according to the standard phenol-chloroform methods, and was diluted to a final concentration of 50 ng/μL.

We selected 8 top associated SNPs (rs10116277, rs1333040, rs10757274, rs2891168, rs2383207, rs4977574, rs10757278, and rs1333049) within the 9p21.3 region that were associated with CAD/MI in previous GWASs (12, 20–23), particularly those with a significant interaction with DM (9, 10, 24). The 8 SNPs were genotyped using matrix-assisted laser desorption/ionization time-of-flight mass spectrometry (MALDI-TOF MS) based on the MassARRAY system (Sequenom, San Diego, CA, USA) according to the manufacturer's protocol. The call rates of all the SNPs were ≥98%.

### Assessment of covariates

Hypertension was defined as a systolic blood pressure ≥140 mmHg, a diastolic blood pressure ≥90 mmHg, or the use of anti-hypertensive drugs. DM was defined as a fasting blood glucose ≥7.0 mmol/L, a non-fasting glucose ≥11.1 mmol/L, or the use of glucose lowering medication (25). Cigarette smoking was defined as smokers or non-smokers and alcohol drinking was defined as drinkers or non-drinkers.

Serum biochemical parameters including triglyceride (TG), total cholesterol (TC), high density lipoprotein cholesterol (HDL-C), and low density lipoprotein cholesterol (LDL-C) were measured before the use of lipid-modifying drugs in the central laboratory of the Ruijin Hospital using an automatic analyzer (Beckman CX-7 Biochemical Auto-analyzer; Brea, CA). Enzymatic methods were used to measure TG and TC concentrations and the phosphotungstic acid/MgCl_2_ method was used to quantify HDL-C and LDL-C. The inter-assay coefficient of variation for all the above lipids was <10%.

### Statistical analysis

Genotype distributions at the 9p21.3 locus were tested for compliance with Hardy Weinberg Equilibrium (HWE) and compared between the case and control groups using Fisher's exact tests. The linkage disequilibrium pattern within the chromosome 9p21.3 locus was examined in 927 control subjects with normal coronary arteries using the SHEsis platform (http://analysis.bio-x.cn/myAnalysis.php) (26). To increase the statistical power, the tag SNPs were selected to construct the GRS based on the following criteria: (1) minor allele frequency (MAF) of > 5%; (2) *r*^2^-value of linkage disequilibrium <0.80; and (3) *P* value of HWE > 0.05. We found that there were three blocks of 9p21.3 locus in strong linkage disequilibrium (*r*^2^ ≥ 0.96): block1 (rs10116277 and rs1333040); block2 (rs10757274, rs4977574 and rs2891168); and block3 (rs10757278 and rs1333049). Four tag SNPs (rs1333040, rs4977574, rs2383207 and rs1333049) were selected to represent each block and construct the GRS. The weighted GRS of each participant was calculated by multiplying the risk alleles at each tag SNP by the risk estimate reported in previous GWASs, as described previously (19, 27). The range of unweighted and weighted GRSs were from 0 to 8.

Odds ratios (ORs) and 95% confidence intervals (CIs) of the 9p21.3 variants for DM and MI, were estimated using a multivariable logistic regression model. The following covariates that were known or suspected to be associated with MI were included in the models: age, sex, smoking and drinking habits, hypertension, diabetes, and circulating lipids concentrations (e.g., TG, HDL-C, and LDL-C). To reduce the confounders due to complex clinical conditions, the 9p21.3-DM association was assessed in 927 people with normal coronary arteries. The significance of the multiplicative interaction between the 9p21.3 locus and DM was tested by adding a cross-product term into the logistic regression model. Departure from the additive model was tested by calculating the relative excess risk due to the interaction (28).

**Figure 1:**
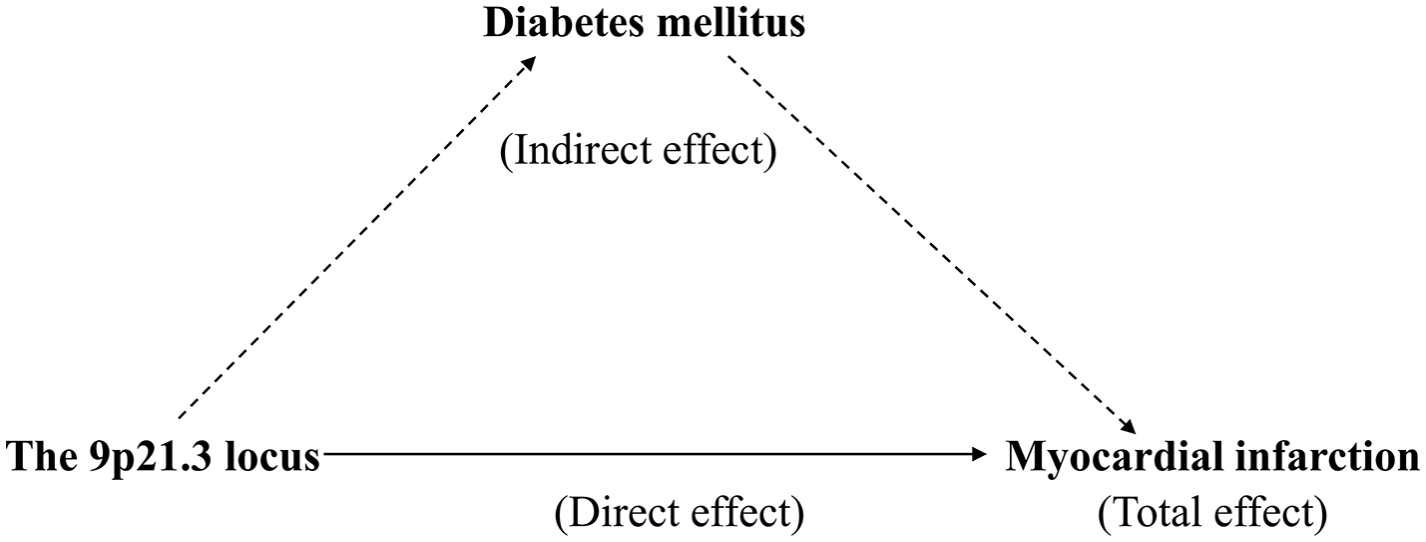
Mediation analysis of the effect of diabetes mellitus (DM) on the association between the 9p21.3 genetic risk score (GRS) and myocardial infarction (MI).

Counterfactual-based mediation analysis was used to test whether DM affects the association between the 9p21.3 locus and MI. All mediation analyses were performed using the Stata Medeff command in 2 steps. First, the binary outcome (MI) was regressed on the main exposure (the 9p21.3 GRS), the proposed mediator (DM), and other relevant covariates. Second, the mediator (DM) was regressed on the exposure variable and the same covariates as in the first step (29, 30). These two regressions were fitted to obtain the effect mediated by DM (indirect effect) and the effect that came about through pathways other than those that involve the mediator (direct effect). The percent mediation was calculated by dividing the natural logarithm of the indirect effect by the natural logarithm of the total effect (Figure 1).

Given age, sex, cigarette smoking and other concomitant diseases (e.g., hypertension) may confound the mediating effect of DM, sensitivity analyses were performed by restricting data in men, elderly people, and individuals without hypertension or smoking habit.

A two-tailed *P* ≤ 0.05 was regarded as statistically significant. All statistical analyses were performed using STATA software version 13.0 (College Station, TX, USA).

## Results

The clinical characteristics of the study participants are summarized according to the case and control groups in Table 1. Individuals who suffered MI were more likely to be smoker, and to have DM and lower HDL-C concentrations, as compared with controls (Table 1). The distribution of the 9p21.3 genotype did not deviate from the HWE (*P* > 0.05) (Supplementary Table 1).

**Table 1:** Baseline characteristics of individuals with myocardial infarction and control subjects.

**Characteristics**	**Myocardial infarction**	**Controls**	***p*-value**
Number	865	927	
Age, years^*^	60.9 ± 10.7	60.1 ± 9.42	0.10
Men, %	80.2	76.9	0.09
TG, mmol/L	1.75 ± 0.96	1.80 ± 1.19	0.30
TC, mmol/L	4.48 ± 1.07	4.43 ± 0.92	0.32
HDL-C, mmol/L	1.06 ± 0.26	1.19 ± 0.33	<0.0001
LDL-C, mmol/L	2.71 ± 0.89	2.62 ± 1.15	0.06
Lp(a), g/L	0.24 ± 0.19	0.19 ± 0.17	<0.0001
Drinker, %	14.9	15.5	0.69
Smoker, %	51.9	33.8	<0.0001
Hypertension, %	65.8	68.3	0.26
Diabetes, %	28.1	12.1	<0.0001
Family history of CVD, %	8.09	8.95	0.51

*TG, triglyceride; TC, total cholesterol; HDL-C; high density lipoprotein cholesterol; LDL-C, low density lipoprotein cholesterol; Lp(a), lipoprotein (a); CVD, cardiovascular diseases*.

* *Continuous variables were reported as mean ± standard error*.

**Figure 2:**
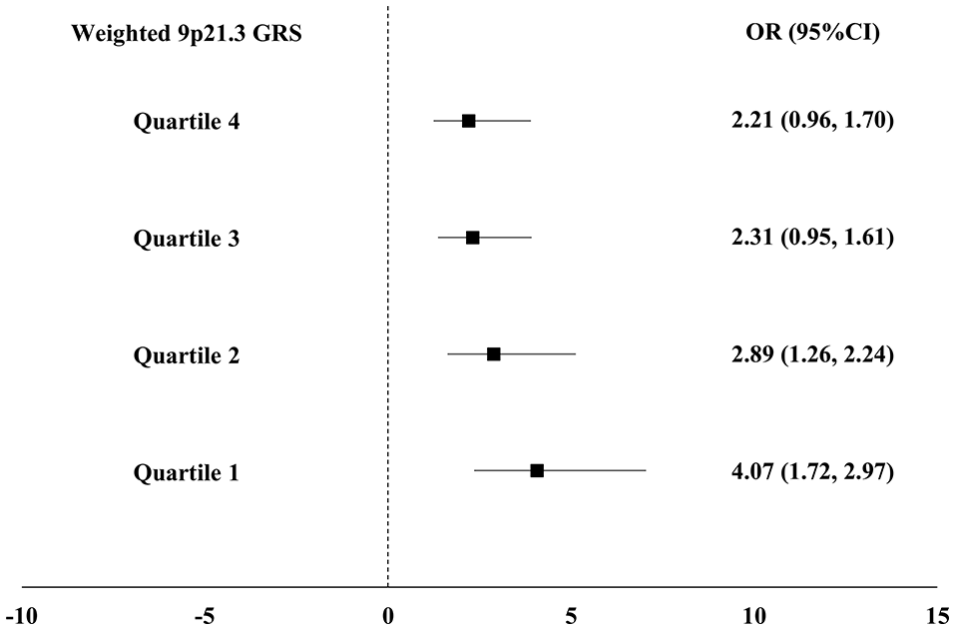
Association between diabetes mellitus (DM) and myocardial infarction (MI) across the weighted 9p21.3 genetic risk score (GRS) quartiles. All models were adjusted for age, sex, smoking and drinking habits, hypertension and serum triglyceride, high density lipoprotein cholesterol, and low density lipoprotein cholesterol concentrations.

As expected, the weighted and unweighted 9p21.3 GRSs were positively associated with both MI and DM after adjustment for conventional risk factors. Each 1 unit increase in weighted 9p21.3 GRS conferred a 9% increase in DM risk (95% CI 1.00–1.19) in the control group and a 14% increase in MI risk (95% CI 1.09–1.20) (Table 2). In addition, DM was consistently associated with MI across the weighted 9p21.3 GRS quartiles. The magnitude of the association between DM and MI exhibited a gradually decrease with increasing 9p21.3 GRS quartiles. The adjusted OR was greatest in the lowest 9p21.3 GRS quartile group (OR 4.07, 95%CI 1.72, 2.97) (Figure 2).

**Table 2:** Association between 9p21.3 genetic risk score and myocardial infarction and diabetes mellitus.

**Variable**	**Crude**		Multivariate adjusted Model^*^	
	**OR (95%CI)**	**P-value**	**OR (95%CI)**	**P-value**
**MYOCARDIAL INFARCTION**				
Unweighted GRS	1.16 (1.11, 1.21)	<0.0001	1.15 (1.10, 1.20)	<0.0001
Weighted GRS	1.16 (1.11, 1.20)	<0.0001	1.14 (1.09, 1.20)	<0.0001
DIABETES MELLITUS^**^				
Unweighted GRS	1.10 (1.01, 1.20)	0.03	1.09 (1.00, 1.19)	0.05
Weighted GRS	1.10 (1.01, 1.20)	0.03	1.09 (1.00, 1.19)	0.049

*OR, odds ratio; CI, confidence interval; GRS, genetic risk score*.

* *All models were adjusted for age, sex, smoking and drinking habits, hypertension, diabetes mellitus, and serum triglyceride, high density lipoprotein cholesterol, and low density lipoprotein cholesterol concentrations*.

** *The analyses were conducted in individuals with normal coronary arteries*.

In the mediation analysis, the models for MI and DM were combined to calculate the indirect effect explained by DM and the direct effect exerted via other pathways (31). There was a 4.40% (95%CI 3.42, 6.52) of the total effect of the weighted 9p21.3 GRS on MI risk mediated by DM. This result was similar to that of unweighted GRS. In the sensitivity analyses, the proportion of MI risk mediated by DM increased to 10% (95%CI 7.30, 18.1) in individuals aged >65 years and to 17.9% (95%CI 12.4, 36.8) among non-smokers (Table 3).

**Table 3:** Mediation analysis of the association between the 9p21.3 genetic risk score and myocardial infarction risk mediated by diabetes mellitus.

**Variables**	Direct effect^*^ OR (95%CI)	**Indirect effect OR (95%CI)**	**Total effect OR (95%CI)**	**Percentage mediated by DM (95%CI), (%)**
Unweighted GRS	1.06 (1.04, 1.08)	1.00 (0.99, 1.01)	1.07 (1.05, 1.09)	4.45 (3.48, 6.56)
Weighted GRS	1.06 (1.04, 1.08)	1.00 (0.99, 1.01)	1.07 (1.05, 1.09)	4.40 (3.42, 6.52)
SENSITIVITY ANALYSES^**^				
> 65 years	1.07 (1.04, 1.11)	1.01 (1.00, 1.02)	1.08 (1.05, 1.12)	10.0 (7.30, 18.1)
Men	1.07 (1.05, 1.09)	1.00 (1.00, 1.01)	1.08 (1.05, 1.10)	6.03 (4.78, 8.55)
Non-hypertension	1.06 (1.03, 1.10)	1.00 (0.99, 1.01)	1.07 (1.03, 1.10)	3.57 (2.40, 7.93)
Non-smoker	1.04 (1.02, 1.06)	1.00 (1.00, 1.02)	1.05 (1.02, 1.08)	17.9 (12.4, 36.8)

*OR, odds ratio; CI, confidence interval; DM, diabetes mellitus; GRS, genetic risk score*.

* *All models were adjusted for age, sex, smoking and drinking habits, hypertension, and serum triglyceride, high density lipoprotein cholesterol, and low density lipoprotein cholesterol concentrations*.

** *Sensitivity analyses were conducted using the weighted genetic risk score*.

**Figure 3:**
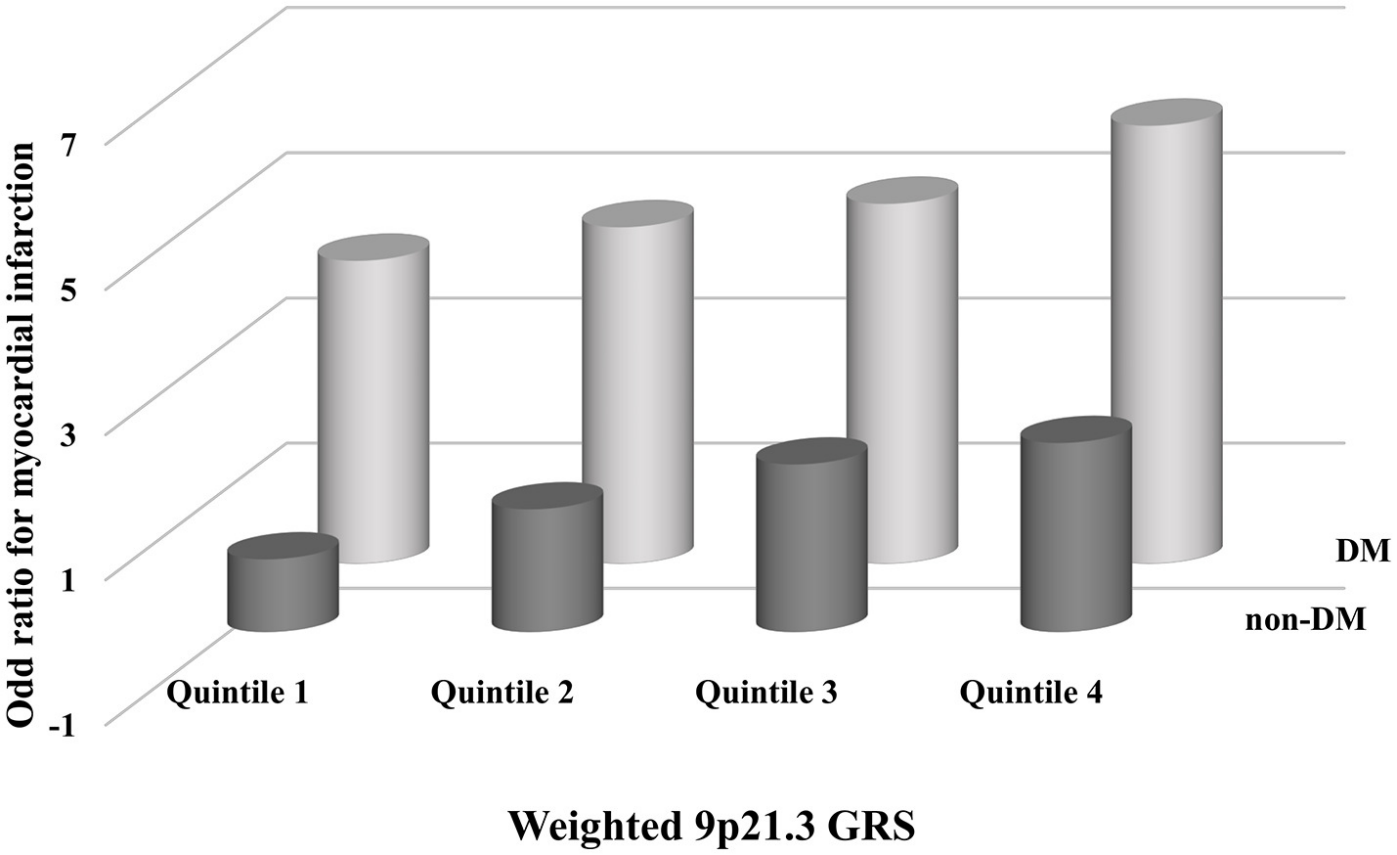
Adjusted odds ratios for myocardial infarction (MI) with combined subgroups of diabetes mellitus (DM) and the 9p21.3 genetic risk score (GRS). Odds ratios were calculated in each subgroup using the lowest quartile of weighted 9p21.3 GRS and non-DM as the reference. All models were adjusted for age, sex, smoking and drinking habits, hypertension and serum triglyceride, high density lipoprotein cholesterol, and low density lipoprotein cholesterol concentrations.

The interaction between GRS and DM was not significant on a multiplicative scale (*P* = 0.06) or an additive scale (*P* = 0.97). Individuals in the highest quartile of weighted 9p21.3 GRS and DM had a significantly increased risk of MI (OR 6.03, 95%CI 3.48, 10.5) compared to those in the lowest quartile of weighted GRS and non-DM (Figure 3).

## Discussion

In this current study based on a Southern Chinese cohort, we used a mediation model to examine whether DM acts as a mediator in the association between the 9p21.3 GRS and MI. The 9p21.3 GRS, significantly associated with MI and marginally associated with DM, had a highly significant direct effect on MI risk. However, only a small fraction of MI risk due to the 9p21.3 locus (indirect effect) could be explained by DM.

Previous studies suggested that the genetic background may play an important role in the predisposition to both metabolic diseases and atherosclerotic CVD (2, 32). The contribution of epigenetics to the etiology of atherosclerosis suggests that advanced statistical models could be important to precisely assess the effect of multifactorial disorders including complex gene-environment interactions and the modifying effect of the intermediate phenotype (mediator) on diseases. Mediation analysis, previously used in quantitative social science and psychology research, is a promising approach to identify the possible mechanisms that mediate causal gene-disease associations. It is flexible to interpretation the extent to which the effect of independent variables on outcomes can be mediated by a potential mediator of interest. It also allows potential bias due to the exposure-mediator interactions to be assessed, which is hard to be addressed using traditional approaches (33).

To the best of our knowledge, this is the first study to examine the DM-dependent and -independent MI risk that is attributable to the 9p21.3 locus. The small mediating effect of DM and the non-significant DM-9p21.3 interaction in the current study suggests that the pathological processes involved in these two major disease loci may be mutually independent. The genetic background favorable to the development of CVD events differ from the genetic background favorable to metabolic diseases, although they may share a common pathway.

There were no significant multiplicative or additive interactions between DM and the 9p21.3 GRS on MI risk. However, the sensitivity analyses revealed that an indirect effect contributed to the 9p21.3-MI association more in individuals aged >65 years and non-smokers. Although we cannot exclude the possibility of chance findings, this observation could be also interpreted as suggesting that elderly people are more likely to suffer diabetic macrovascular diseases. The enhanced mediating effect of DM in non-smokers may be explained by the fact that cigarette smoking is a sufficient cause of CAD/MI itself, which may overwhelm the effect of genetics and DM on the risk of CAD to some extent. This notion is supported by previous genetic studies reporting that the increased CAD risk caused by the 9p21.3 risk alleles was attenuated in smokers (34, 35).

Although the 9p21.3-MI association is not principally mediated by DM, it could be significantly reinforced in the presence of DM, as reflected by a strong synergistic effect of the 9p21.3 GRS and DM in determining the risk of MI. The mechanism by which DM could enhance the MI risk via genetic factors remains unknown. It is plausible that DM increases the individuals' vulnerability to MI events based on a greater atheromatous burden derived from the high genetic risk of the 9p21.3 locus. This notion is supported by a previous prospective study that found that poor glycemic control was significantly associated with increased MI risk due to the 9p21.3 risk alleles in diabetic people (10).

The strength of the current study is using a mediation analysis that goes beyond traditional analyses to provide a more causal interpretation of gene-environment interactions for a certain assumption (30). The use of GRS substantially increases the statistical power and represents the function of the 9p21.3 locus better than a single SNP. All participants underwent coronary angiography, allowing us to avoid misclassifying asymptomatic CAD and other cardiac disorders (e.g., pericarditis and cardiomyopathy) that may exhibit similar symptoms as MI.

The current study has several limitations. First, we only examined some of the 9p21.3 variants and traditional cardiovascular risk factors, and thus could not exclude the influence of unmeasured mediator-outcome confounders or interactions between the exposure and mediator that may play a role in observational studies. Some other factors (e.g., physical inactivity or obesity) could affect DM and MI in the same direction, which would result in underestimation of the direct effect and overestimation of indirect effects. Second, the GRS only included four 9p21.3 GRS and so may be not representative of all functional SNPs of the 9p21.3 locus. Some DM-related 9p21.3 variants, which were yet to be identical with CAD/MI in GWASs, were not included in our study. Thus, the 9p21.3-DM association and the mediating effect of DM on 9p21.3-MI association might be underestimated. Further GWASs identifying more 9p21.3 variants related to CAD/MI and/or DM are necessary to improve the predictive capability of the 9p21.3 GRS. Third, the study included mostly individuals of Southern Chinese Han ethnicity, and so it is unclear whether these findings could be generalized to other populations. Therefore, the study should be replicated in a cohort with a larger sample size to confirm the current results.

In conclusion, the associations between the 9p21.3 variants and MI could be explained by DM to a small extent. Mediation analysis provides novel insights into the mechanisms by which gene-environment interactions affect diseases. Mechanisms independent of DM may explain a high proportion of the 9p21.3-MI association. Further studies providing additional physiological insights into the synergistic mechanisms of these two common chronic diseases may help to interpret our findings.

## Ethics statement

The study protocol was approved by the ethics committee of the Ruijin Hospital, Shanghai Jiao Tong University School of Medicine and followed the norms of the World's Association Declaration of Helsinki. All participants provided written informed consent at the time of enrollment.

## Author contributions

ZW, HS, and WJ conceived the study. ZW, XS, and XG analyzed the data. ZW, LL, XG, and WJ interpreted the results. ZW and WJ drafted the manuscript. All authors approved the contents of the manuscript.

### Conflict of interest statement

The authors declare that the research was conducted in the absence of any commercial or financial relationships that could be construed as a potential conflict of interest.
